# Monitoring Early Response to Anti-Angiogenic Therapy: Diffusion-Weighted Magnetic Resonance Imaging and Volume Measurements in Colon Carcinoma Xenografts

**DOI:** 10.1371/journal.pone.0106970

**Published:** 2014-09-15

**Authors:** Moritz Jörg Schneider, Clemens Christian Cyran, Konstantin Nikolaou, Heidrun Hirner, Maximilian F. Reiser, Olaf Dietrich

**Affiliations:** 1 Josef Lissner Laboratory for Biomedical Imaging, Institute for Clinical Radiology, Ludwig-Maximilians-University Hospital Munich, Munich, Germany; 2 Laboratory for Experimental Radiology, Institute for Clinical Radiology, Ludwig-Maximilians-University Hospital Munich, Munich, Germany; 3 Department of Diagnostic and Interventional Radiology, University Hospital Tübingen, Tübingen, Germany; Universidade de São Paulo, Brazil

## Abstract

**Objectives:**

To evaluate the use of diffusion-weighted MRI (DW-MRI) and volume measurements for early monitoring of antiangiogenic therapy in an experimental tumor model.

**Materials and Methods:**

23 athymic nude rats, bearing human colon carcinoma xenografts (HT-29) were examined before and after 6 days of treatment with regorafenib (n = 12) or placebo (n = 11) in a clinical 3-Tesla MRI. For DW-MRI, a single-shot EPI sequence with 9 b-values (10–800 s/mm^2^) was used. The apparent diffusion coefficient (ADC) was calculated voxelwise and its median value over a region of interest, covering the entire tumor, was defined as the tumor ADC. Tumor volume was determined using T2-weighted images. ADC and volume changes between first and second measurement were evaluated as classifiers by a receiver-operator-characteristic (ROC) analysis individually and combined using Fisher's linear discriminant analysis (FLDA).

**Results:**

All ADCs and volumes are stated as median±standard deviation. Tumor ADC increased significantly in the therapy group (0.76±0.09×10^−3^ mm^2^/s to 0.90±0.12×10^−3^ mm^2^/s; p<0.001), with significantly higher changes of tumor ADC than in the control group (0.10±0.11×10^−3^ mm^2^/s vs. 0.03±0.09×10^−3^ mm^2^/s; p = 0.027). Tumor volume increased significantly in both groups (therapy: 347.8±449.1 to 405.3±823.6 mm^3^; p = 0.034; control: 219.7±79.5 to 443.7±141.5 mm^3^; p<0.001), however, the therapy group showed significantly reduced tumor growth (33.30±47.30% vs. 96.43±31.66%; p<0.001). Area under the curve and accuracy of the ADC-based ROC analysis were 0.773 and 78.3%; and for the volume change 0.886 and 82.6%. The FLDA approach yielded an AUC of 0.985 and an accuracy of 95.7%.

**Conclusions:**

Regorafenib therapy significantly increased tumor ADC after 6 days of treatment and also significantly reduced tumor growth. However, ROC analyses using each parameter individually revealed a lack of accuracy in discriminating between therapy and control group. The combination of both parameters using FLDA substantially improved diagnostic accuracy, thus highlighting the potential of multi-parameter MRI as an imaging biomarker for non-invasive early tumor therapy monitoring.

## Introduction

Monitoring the response to anti-cancer treatment is an integral part of oncology. With the introduction of novel molecular cancer therapies to clinical routine it has become apparent that conventional, solely morphology-based imaging criteria, such as the Response Evaluation Criteria in Solid Tumors (RECIST) [1], provide limited sensitivity to assess therapy response, particularly during initial treatment [2], [3]. Technical developments in recent years introduced a variety of new functional imaging methods, such as diffusion-weighted MRI (DW-MRI) or perfusion imaging. These new methods complement established morphological information and are also applicable as in-vivo imaging biomarkers of therapy response for monitoring of anti-cancer treatment.

DW-MRI is a method to visualize and quantify the mobility of water molecules in the observed tissue [4], [5]. The thermally driven random motion, the so-called Brownian motion, is influenced by the properties of the surrounding tissue microstructure, e. g. cellular density and cell integrity. While qualitative DW-MRI is already widely used in oncology for the detection of metastases, several recent reviews have highlighted the potential of quantitative DW-MRI, i.e. the measurement of the apparent diffusion coefficient (ADC), to predict and to monitor response to anti-cancer treatment [6]–[11]. Generally, malignant lesions are known to exhibit lower ADCs compared to healthy tissue and benign lesions, which is mainly a result of the commonly higher cellularity of malignancies [12]–[17]. On the other hand, studies measuring pre-treatment ADCs have found, that relatively high initial ADCs in malignant lesions were predictive of poor therapy outcome [18]–[21], while increasing ADCs over the duration of various anti-cancer treatments were associated with therapy response in malignant breast metastases [22], rhabdomyosarcomas [23], prostate carcinoma xenografts [24], colorectal liver metastases [25] and cholangiocarcinomas [26].

The novel oral multi-kinase inhibitor regorafenib exhibits anti-angiogenic and anti-proliferative effects on glioblastoma, breast, and renal cell carcinoma xenografts [27] and is clinically approved for treatment in metastatic colorectal cancer [28]. Pharmacologically, regorafenib belongs to the group of multi tyrosinekinase inhibitors and inhibits multiple membrane-bound and intracellular kinases involved in tumorigenesis, neoangiogenesis and in the preservation of the tumor microenvironment. *In vitro*, regorafenib has been shown to inhibit the activity of VEGFR-1, VEGFR-2, VEGFR-3, PDGFR-α, PDGFR-β, FGFR-1, FGFR-2, RET, KIT, TIE2, DDR2, TrkA, Eph2A, RAF-1, BRAF, SAPK2, PTK5 and Abl at concentrations that can be achieved clinically [27].With reported pro-apoptotic effects of regorafenib in colon carcinoma xenografts [29] and studies reporting significant positive correlations between ADCs and the number of apoptotic tumor cells [30], we hypothesized that quantitative ADC measurements would be applicable to sensitively assess the effects of regorafenib in an experimental model of human colon cancer. An anticipated potential limitation of this approach as reliable imaging biomarker is necrotic tumor transformation with progressing tumor growth. Necrotic transformation also leads to augmented water mobility [15] and potentially impairs the specificity of ADC measurements for assessing treatment response. A meaningful combined evaluation of tumor morphology and DW-MRI could reduce the individual limitations of each approach, allowing for non-invasive response monitoring during initial treatment. Such a combination is constituted by Fisher's linear discriminant analysis (FLDA) [31], which has been demonstrated to increase the accuracy to separate between malignant and benign lesions in the vertebral bone marrow by incorporating ADC and *T*_2_ relaxation time values compared to using each classifier individually [32].

The purpose of this study was to evaluate quantitative DW-MRI and tumor growth measurements, individually and combined using a discriminant analysis approach, as means of distinguishing between therapy and control group of human colorectal carcinoma in rats under regorafenib or placebo therapy. We hypothesized that the combination of both approaches outperforms each classifier individually and can be used to monitor anti-angiogenic therapy non-invasively.

## Materials and Methods

### Animal Model

This study was approved by the Government of Upper Bavaria Committee for Animal Research (Gz.55.2-1-54-2532-33-10) and was carried out in accordance with the guidelines of the National Institute of Health for the care and use of laboratory animals. For the experiments twenty-three female athymic rats (7–8 weeks old, Harlan Laboratories Inc., Indianapolis, IN) were used. 2×10^6^ cells of the human colon carcinoma cell line HT-29 (ATCC HTB-38) suspended in a total volume of 0.5 mL as a 1∶1 mixture of phosphate buffered saline pH 7.4 (PBS) and Matrigel (BD Biosciences, San Jose, CA) were injected subcutaneously into the left flanks. Prior to MRI the xenografts were allowed to grow to a reasonable size for imaging of approximately 400 mm^3^ (assessed by daily caliper measurements in three dimensions (*a*×*b*×*c*×0.5)) and the animals were randomly assigned to either the therapy (*n* = 12) or the control group (*n* = 11). After the initial MRI on day 0 the animals and were treated daily for one week with the multi-tyrosine kinase inhibitor, regorafenib (Bayer HealthCare, Leverkusen, Germany), respectively with the placebo. On day 7 a follow-up MRI was performed to assess the effects of regorafenib on tumor growth and the ADC, after which the animals were euthanized via intracardiac injection of potassium chloride.

### Tumor Therapy

The therapy group was administered 10 mg/kg body weight of regorafenib daily, formulated as a solution in polypropylene glycol/PEG400/Pluronic F68 (42.5/42.5/15 + 20% Aqua), via gastric gavage, using a dedicated 16-gauge curved buttoned cannula. The control group received volume-equivalent applications of the regorafenib solvent daily.

### MR Image Acquisition

Prior to MR imaging, animals were anaesthetized with isoflurane (5% for induction, 2.5% for maintenance, administered in pure oxygen). Scans were conducted on a clinical 3-Tesla whole-body MRI system (MAGNETOM Verio, Siemens Healthcare, Erlangen, Germany) with a small 4-channel flex coil (Siemens Healthcare, Erlangen, Germany).

DW-MRI was performed using a diffusion-weighted single-shot spin-echo sequence with echoplanar imaging (EPI) readout. A modified monopolar diffusion encoding scheme [33] was used to achieve a reduction in TE and therefore an increase in signal intensity. Trace diffusion-weighted images were calculated by averaging images obtained with diffusion gradients in 3 orthogonal directions. A total of 9 diffusion weightings (b-values) were acquired (*b* = 10; 25; 50; 80; 130; 200; 350; 550; 800 s/mm^2^) with the parameters listed in Table 1. To assess the tumor volumes, a *T*_2_-weighted turbo-spin-echo sequence with a high in-plane resolution of 0.3×0.3 mm^2^ was used (Table 1).

**Table 1:** MRI acquisition parameters.

Parameter	DW-MRI	T2-weighted MRI
Acquisition plane	Axial	Axial
Repetition time (ms)	2500	9560
Echo time (ms)	55	91
Signal averages	8	3
Acquisition matrix	68×52	192×192
Reconstructed matrix	136×104	192×192
Field of view (mm^2^)	65×50	60×60
Slice thickness (mm)	2	1.5
Slice gap (mm)	0.4	0
Number of slices	12	35
Parallel imaging factor	2 (GRAPPA)	2 (GRAPPA)
Fat supression	On	Off
b-values (s/mm^2^)	10, 25, 50, 80, 130, 200, 350, 550, 800	-
Acquisition time (min)	10:08	6:53

### Image Analysis

Image analysis was performed on a dedicated workstation using our in-house software PMI (Platform for Research in Medical Imaging) [34] written in IDL (ITT Visual Information Systems, Boulder, CO).

#### DW-MRI

**Figure 1:**
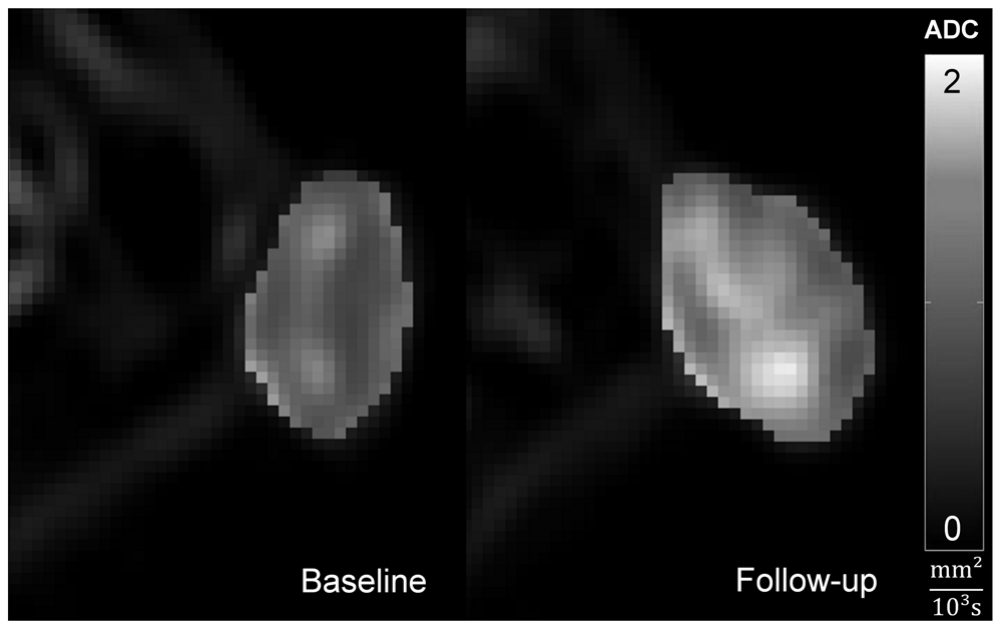
Voxelwise calculated ADC maps in the subcutaneous colon carcinoma of a therapy animal before and after 6 days of therapy with regorafenib laid over diffusion-weighted images with b = 10 s/mm^2^. The ADC maps display a prominent increase, which is also reflected in the median tumor ADC value for this animal: ADC_B_ = 0.762×10^−3^mm^2^/s at day 0, ADC_F_ = 1.137×10^−3^mm^2^/s at day 7.

Prior to quantitative analysis, the diffusion-weighted images were rigidly registered along the b-value-dimension using a Fourier cross-correlation method to keep bulk-motion from affecting the diffusion coefficients. The ADC of each voxel was calculated by non-linear least-squares fitting of the measured signal intensities from all acquired b-values to the monoexponential diffusion model: *S(b)*  = *S_0_*× exp(− *b*×*ADC*), where *b* is the b-value and *S*_0_ the signal intensity at *b* = 0 (Figure 1).

**Figure 2:**
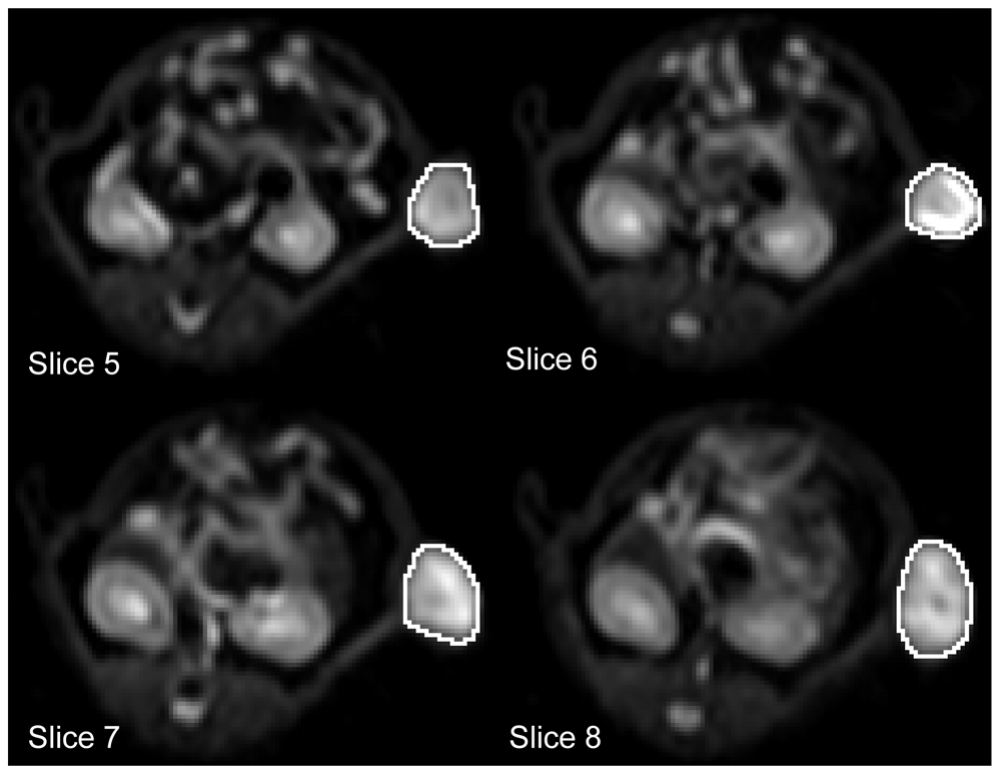
Region of interest (ROI) placement on diffusion-weighted images with b = 10 s/mm^2^ over 4 example slices to calculate the median tumor ADC. Total ROI extends over 10 slices for this measurement.

Obtaining quantitative parameters from MRI measurements is highly dependent on region of interest (ROI) placement, which often suffers from poor reproducibility. To obtain robust results and to minimize subjective influences on the ROI definition, we defined a 3D volume of interest (VOI) covering the entire tumor on multiple slices of the diffusion-weighted data for each animal and measurement (Figure 2). The median of the ADC distributions inside the VOIs were then taken as the representative tumor ADCs for statistical analysis. The tumor ADCs are denoted ADC_B_ and ADC_F_ for the baseline and follow-up measurements, respectively.

#### Volume Measurement

**Figure 3:**
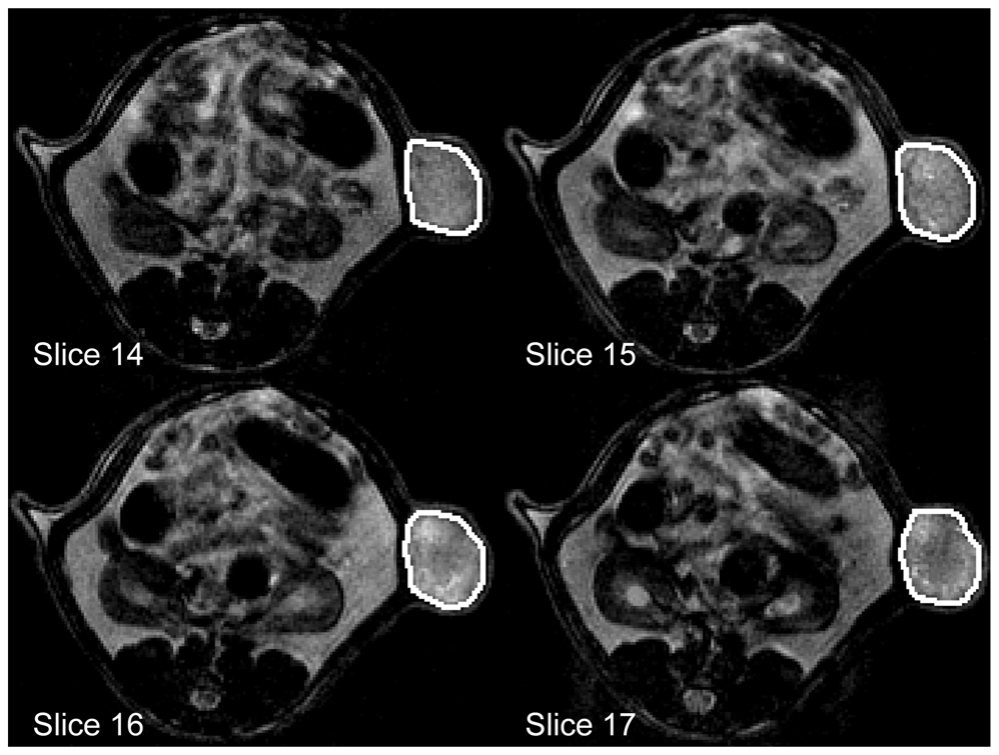
Region of interest (ROI) placement on *T*_2_-weighted images over 4 example slices to measure the tumor volume. Total ROI extends over 22 slices for this measurement.

The tumor volumes were determined based on the morphologic *T*_2_-weighted images, which allowed for a clear delineation of the subcutaneous xenografts. For each animal and each measurement, a VOI was placed over several slices to cover the entire tumor (Figure 3). The combined volume of all voxels inside each VOI was defined as the tumor volume (the slice gap is 0 mm and therefore did not need to be taken into account) and denoted as VOL_B_ and VOL_F_ for the baseline and follow-up measurements, respectively. To accommodate for varying pre-therapy tumor sizes, tumor growth was assessed in percentages relative to the baseline value rather than absolute growth.

### Statistical Analyses

All statistical analyses were performed using the statistical computing language R [35]. Within each group, median values and standard deviations of the evaluated parameters over all animals were determined. For intragroup comparison between baseline and follow-up parameters, the paired Wilcoxon signed-rank test was used. The comparison of the parameters between the therapy and control group was performed using the non-paired Mann-Whitney U test. The observed changes for both tumor ADCs (ΔADC) and volumes (ΔVOL) from baseline to follow-up were also used for analyses. To evaluate diagnostic performance of either ΔADC or ΔVOL in discriminating therapy from control group, the receiver operating characteristics (ROC) curves were analyzed using the R package pROC [36]. Parameters of interest were the area under the curve (AUC) as well as the optimal threshold and the resulting sensitivity, specificity, and diagnostic accuracy. The statistical significance of the difference between the AUCs was determined using the method as described by DeLong et al. [37] based on generalized U-statistics to generate an estimated covariance matrix.

Additionally, for each group the linear correlation between ΔADC and ΔVOL was determined using Pearson's product-moment correlation. To assess if the combination of both parameters, ΔADC and ΔVOL, increases the ability to distinguish between therapy and control group compared to the individual classifiers, Fisher's linear discriminant analysis [31] was performed using the R package Bioconductor [38], [39]. FLDA is a statistical method used to find a linear combination of given classifiers, in our case ΔADC and ΔVOL, which allows for an optimal separation of a group of classes. The result from the determined linear combination was then again used for statistical comparison between the two groups and as a classifier to perform a ROC curve analysis. For all analyses, p-values of less than 0.05 were considered statistically significant.

## Results

### Tumor ADC

**Figure 4:**
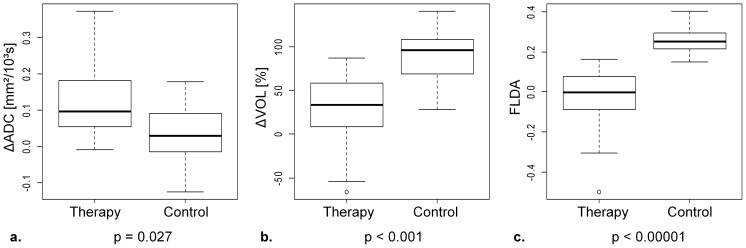
Box plots (first, second, and third quartile, range and outlier) of (a) ΔADC, (b) ΔVOL and (c) the results from the linear combination calculated with Fisher's linear discriminant analysis (FLDA) for each group and the corresponding p-value for the difference between them. Although significantly different, ΔADC and ΔVOL display distinctive overlaps between the two groups. The result from FLDA demonstrates a marked improvement in the group discrimination with nearly no overlap, resulting in a highly significant difference.

**Figure 5:**
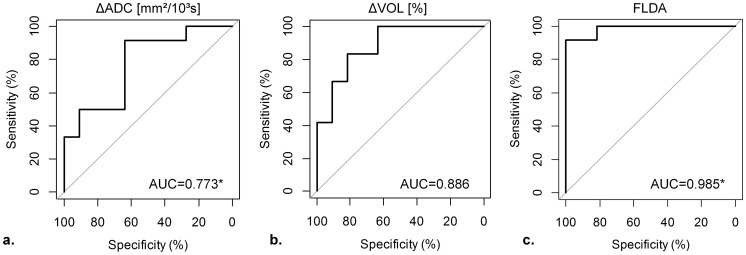
ROC curve analysis using (a) ΔADC, (b) ΔVOL and (c) the result from the linear combination calculated with Fisher's linear discriminant analysis (FLDA) to illustrate performance in differentiating therapy from control group. The combined approach using FLDA outperforms the use of ΔADC and ΔVOL notably. Optimal sensitivity and specificity for each parameter and the corresponding thresholds are summarized in Table 3. * AUC using ΔADC vs AUC using FLDA: p = 0.035.

Median tumor ADCs with standard deviations and the results from the statistical analyses are summarized in Table 2 (see Table S1 for voxelwise calculated ADC distributions and Table S2 for individual tumor ADC values (median of the distributions) for each animal and measurement). A statistically highly significant (p<0.001) difference in tumor ADC between baseline and follow-up measurement was found in the therapy group, where the median increased from ADC_B_ = 0.782(±0.085)×10^−3^ mm^2^/s to ADC_F_ = 0.911(±0.121)×10^−3^ mm^2^/s. Conversely, no significant alteration was found in the control group (ADC_B_ = 0.740(±0.087)×10^−3^ mm^2^/s to ADC_F_ = 0.770 (±0.070)×10^−3^ mm^2^/s, p = 0.24). Statistically significant differences between the two groups were found for the follow-up tumor ADCs (p<0.001) as well as for the observed changes in tumor ADCs (therapy: ΔADC = 0.130(±0.110)×10^−3^ mm^2^/s; control: ΔADC = 0.030(±0.087)×10^−3^ mm^2^/s; p = 0.268; Figure 4a). The ROC curve analysis for ΔADC is illustrated in Figure 5a. The AUC was 0.773 and using an optimal threshold of ΔADC = 0.329×10^−3^ mm^2^/s (above = therapy), a sensitivity of 91.7%, a specificity of 63.6%, and a diagnostic accuracy of 78.3% in differentiating between therapy and control group based on ADC changes was obtained (Table 3).

**Table 2:** Median tumor ADCs ± standard deviation for each measurement and group.

Group	ADC_B_ (10^−3^mm^2^/s)	ADC_F_ (10^−3^mm^2^/s)	ΔADC (10^−3^mm^2^/s)
Therapy	0.76±0.09*	0.90±0.12* §	+0.10±0.11†
Control	0.73±0.09	0.75±0.07§	+0.03±0.09†

Note: ADC_B_: baseline tumor ADC; ADC_F_: follow-up tumor ADC, ΔADC: tumor ADC changes between measurements.

*Therapy ADC_B_ vs. therapy ADC_F_: p<0.001.

§ Therapy ADC_F_ vs. control ADC_F_: p<0.001.

† Therapy ΔADC vs. control ΔADC: p = 0.027

**Table 3:** Results from the ROC curve analysis using ΔADC, ΔVOL, and FLDA.

Classifier	AUC	Threshold	Sensitivity	Specificity	Accuracy
ΔADC	0.773*	0.033×10^−3^mm^2^/s	91.7%	63.6%	78.3%
ΔVOL	0.886	+61.35%	81.8%	83.3%	82.6%
FLDA	0.985*	+0.139[a.u.]	91.7%	100%	95.7%

Note: ΔADC: tumor ADC changes between measurements, ΔVOL: tumor volume changes between measurements, FLDA: result from Fisher's linear discriminant analysis, AUC: area under the curve.

*AUC using ΔADC vs. AUC using FLDA: p = 0.035.

### Tumor Volume

**Figure 6:**
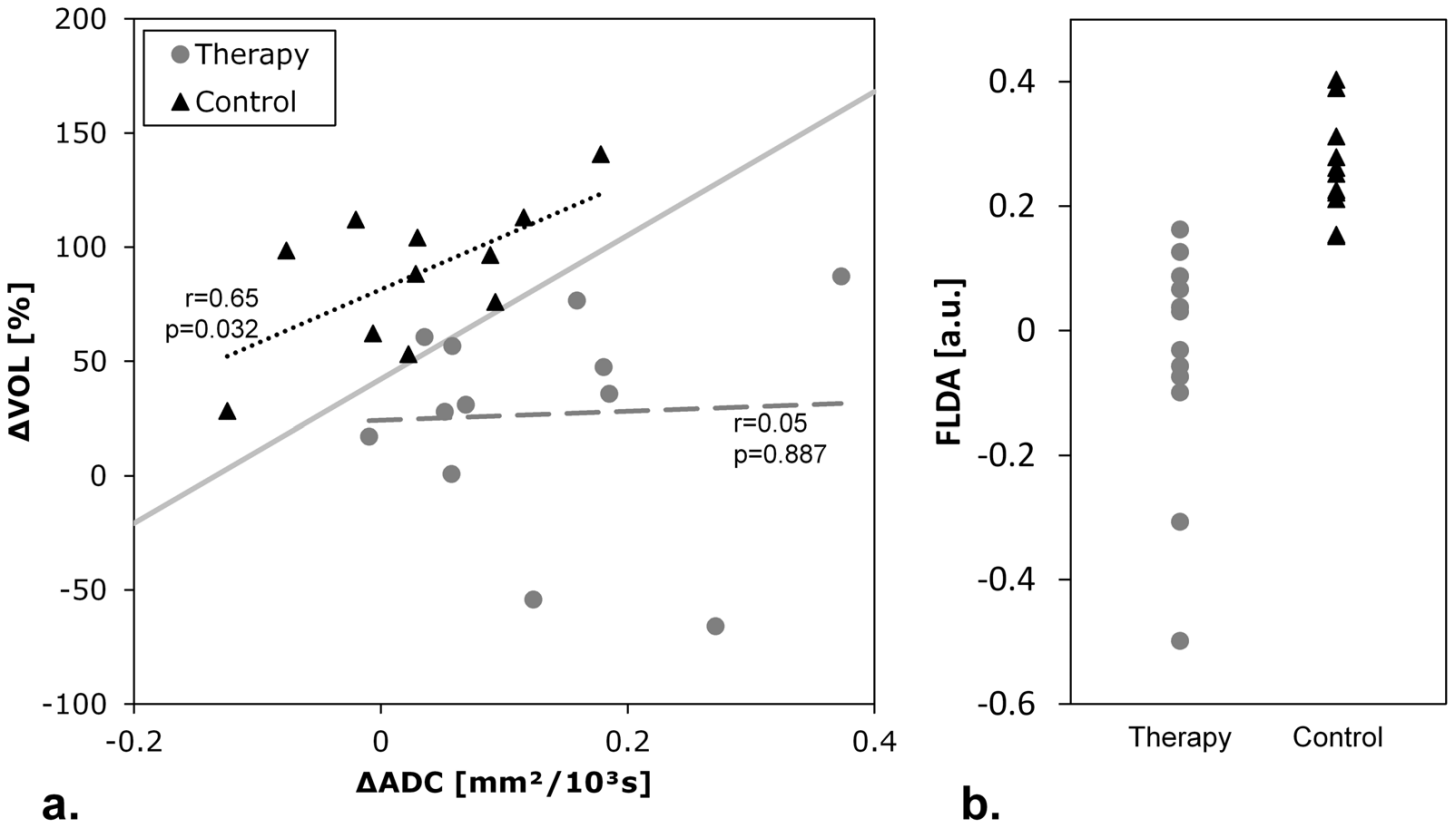
Fisher's linear discriminant anaylsis of volume and ADC data. Panel (a) illustrates thescatterplot of ΔVOL vs. ΔADC for each tumor. The solid grey line represents the optimal threshold determined by the ROC curve analysis; the linear regressions for each group (dashed line for therapy, dotted line for control) are annotated with Pearson's correlation coefficient r and p-value. (b) Results from the FLDA-derived linear combination of ΔADC and ΔVOL (FLDA = 0.0033×ΔVOL[%] - 1.0366×ΔADC[10^−3^ mm^2^/s]).

Median tumor volumes with standard deviations and the results from the statistical analyses are summarized in Table 4 (see Table S3 for individual tumor volumes for each animal and measurement). Both groups displayed a statistically significant increase in tumor volume from baseline to follow-up measurements (therapy: p = 0.034; control: p<0.001), however, the observed relative volume increase in the therapy group (ΔVOL = 33.29(±47.30)%) was significantly smaller than in the control group (ΔVOL = 96.43(±31.66)%); p<0.001; Figure 4b). The ROC curve analysis for ΔVOL is illustrated in Figure 5b. The AUC was 0.886 and using an optimal threshold of ΔVOL = 61.35% (below = therapy) a sensitivity of 81.8%, a specificity of 83.3%, and a diagnostic accuracy of 82.6% in differentiating between therapy and control group based on tumor growth was obtained (Table 3). There was no statistically significant difference between the AUC from ΔADC and ΔVOL (p = 0.419). The control group displayed a moderate correlation between ΔADC and ΔVOL (r = 0.65, p = 0.0319, figure 6a, dotted red line), which was not the case for the therapy group (r = 0.05, p = 0.887, Figure 6a, dashed blue line).

**Table 4:** Median tumor volumes ± standard deviation for each measurement and group.

Group	VOL_B_ (mm^3^)	VOL_F_ (mm^3^)	ΔVOL (%)
Therapy	347.8±449.1*	405.3±823.6*	33.30±47.30†
Control	219.7±79.5§	443.7±141.5§	96.43±31.66†

Note: VOL_B_: baseline tumor volume; VOL_F_: follow-up tumor volume, ΔVOL: tumor volume changes between measurements.

*Therapy VOL_B_ vs. therapy VOL_F_: p = 0.034.

§ Control VOL_B_ vs. control VOL_F_: p<0.001.

† Therapy ΔVOL vs. control ΔVOL: p<0.001.

### Fisher's Linear Discriminant Analysis

The results from FLDA are also shown in Figure 6. The two groups display a statistically highly significant difference (p<0.00001, Figure 4c) between the results from the determined linear combination (FLDA(ΔVOL, ΔADC) = 0.0033×ΔVOL[%] −1.0366×ΔADC[10^−3^mm^2^/s]). The ROC curve analysis for FLDA is illustrated in Figure 5c. The AUC was 0.985 and using an optimal threshold of FLDA = 0.139 (below = therapy) a sensitivity of 91.7%, a specificity of 100%, and a diagnostic accuracy of 95.7% in differentiating between therapy and control group based on FLDA was obtained (Table 3). The AUC yielded by FLDA was significantly larger than the AUC yielded by ΔADC (p = 0.036), however, there was no significant difference compared to the AUC yielded by ΔVOL (p = 0.145).

## Discussion

In this study, we used MRI in an experimental colon carcinoma model to evaluate the influence of the recently FDA-approved multi-kinase inhibitor regorafenib [28] on the water diffusivity in the tumorous tissue and on tumor growth to assess the potential for non-invasive therapy monitoring using ADC and tumor volume measurements. We observed that the one-week treatment significantly increased tumor ADCs as well as significantly reduced tumor growth, however, ROC curve analyses using each parameter individually revealed a lack of accuracy (with values of about 80%) in discriminating between therapy and control group. The combination of both parameters using Fisher's linear discrimination distinctively increased the diagnostic accuracy to more than 95%, thus advocating the capability for therapy monitoring.

### Tumor ADC

Diffusion-weighted MRI revealed microstructural changes in the tumorous tissue reflected by an increased water diffusivity induced by a one-week therapy with regorafenib. The significant increase in tumor ADC in the therapy group is likely due to apoptosis, which was shown to be significantly unregulated by regorafenib therapy in the same tumor cell line (HT29) [29]. A correlation between apoptosis and water diffusivity in tumorous tissue has been observed in various published studies [30], [40]–[42]. Zhang et al. examined mice bearing CT26 colorectal carcinoma tumors under radiotherapy using DW-MRI and histological analysis [40], which led the authors to identifying a significant positive correlation between the percentage of ADC changes and the apoptotic index (TUNEL).

The significant increase in tumor ADCs in the therapy group and more importantly the significant differences in the observed tumor ADC changes between the two groups mark DW-MRI as a potential biomarker for monitoring of molecular cancer therapy, including multi-tyrosine kinase inhibitors such as regorafenib. While Thoeny et al. have reported an initial decrease in water diffusivity in the first hours after anti-cancer therapy initiation [23], an increasing ADC in the tumorous tissue after several days of anti-cancer treatment has widely been associated with therapy response [21]–[23], [26], [43]–[45]. However, in our study the median tumor ADC in the control group also displayed a moderate increase, yet of no statistical significance. The rapid tumor growth and the significant correlation of growth and tumor ADC changes between baseline and follow-up measurement observed in the control group indicate that this increase in water diffusivity is most likely caused by progressing necrotic transformation, which is expected particularly in the untreated control group. Herneth et al. studied ADCs of squamous cell tumors implanted in mice at various tumor sizes [15], reporting that ADCs increased significantly with tumor progression and the areas with increased ADCs correlated well with histologically determined areas of necrosis.

While regorafenib seems to have a significant effect on water diffusivity, the overlap in the observed tumor ADC changes between therapy and control group leads to a less accurate discrimination and therefore to a limitation of the therapy monitoring capabilities of DW-MRI. To increase diagnostic accuracy it is therefore advisable to combine ADC measurements with additional information about tumor progression, such as tumor volume.

### Tumor Volume

The tumor volume increased significantly between baseline and follow-up measurement in both groups. This strongly indicates that the Response Evaluation Criteria in Solid Tumors (RECIST) [1] based on morphologic properties of the target lesion (longest diameter), is not suitable to monitor early response of anti-angiogenic tumor therapy [2], [3]. However, the relative tumor growth was significantly reduced by the regorafenib therapy, as the control group tumors displayed a median relative tumor growth of 96.4% while therapy group tumors only grew by 33.3%. Similar results were previously published by Abou-Elkacem et al. [46], who observed that regorafenib therapy in a murine CT26 metastatic colon cancer model, amongst other anti-angiogenic and anti-metastatic effects, significantly inhibited tumor growth. The ROC curve analysis using relative tumor growth yielded a slight increase in AUC compared to using tumor ADC changes, nevertheless, tumor growth lacks diagnostic accuracy when used as classifier to distinguish between control and therapy group animals after 6 days of regorafenib therapy.

### Fisher's Linear Discriminant Analysis

Fisher's linear discriminant analysis is an effective method to combine two or more classifiers in separation problems [31]. Biffar et al. demonstrated that the use of FLDA to combine ADC and *T*_2_ relaxation times of water in the vertebral bone marrow allowed for increased sensitivity and accuracy in the separation between malignant and benign lesions compared to using each classifier individually [32]. In the present study, a significant correlation between tumor ADC changes and relative tumor growth was found in the control group but not in the therapy group. While this indicates that the observed ADC changes are likely to have different physiological causes, it further promotes the application of a discriminant analysis for the purpose of increased diagnostic accuracy. Accordingly, the combined classifier resulting from FLDA of tumor ADC changes and relative tumor growth improves the discrimination between therapy and control group substantially compared to the individual use. All tumors, except for one false negative (therapy group tumor classified as control group tumor), were classified correctly using the optimal threshold determined by the ROC curve analysis. This result highlights that water diffusivity in tumorous tissue potentially reveals insight on tumor therapy response, but has to be evaluated in a meaningful way with regard to tumor morphology.

### Limitations

The linear combination of ADC and volume changes calculated with FLDA and also the thresholds determined with the ROC curve analyses are specific to the tumor type, the therapy and the time interval between measurements. These parameters may have to be reevaluated according to the respective settings. However, the concept of the method presented in this study to integrate morphological and functional information as complementing parameters should remain valid.

**Figure 7:**
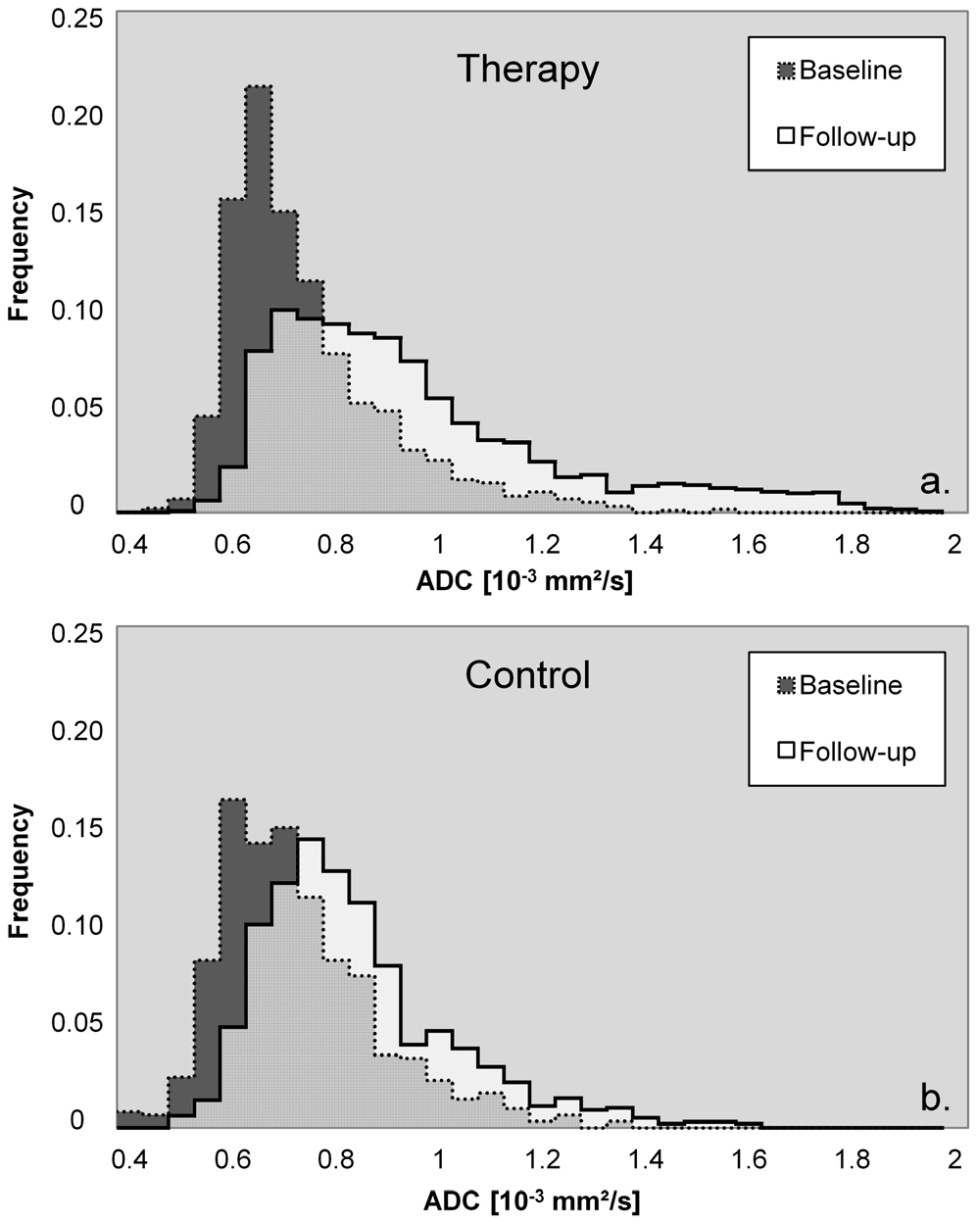
Exemplary histogram distributions of the voxelwise calculated ADCs inside the volume of interest for a (a) therapy and (b) control group animal. The median tumor ADC increased in both cases (therapy: 0.72×10^−3^ mm^2^/s to 0.91×10^−3^ mm^2^/s, control: 0.73×10^−3^ mm^2^/s to 0.82×10^−3^ mm^2^/s), however, the therapy tumor grew by 36%, while the control group tumor grew by 76%.

For further analysis, it may be possible to gain additional insight on the tumor physiology by investigating the histogram shape (e.g. variance or skewness) of the ADC distribution inside the VOI (Figure 7) if the voxel count of the VOI is sufficiently large (i.e. several hundred voxels per VOI). Other possible DW-MRI based evaluations not included in this study are the assessment of intravoxel incoherent motion [47], [48] or diffusional kurtosis parameters [49].

## Conclusions

Using quantitative DW-MRI, we found that therapy of human colon carcinoma xenografts with the multi-tyrosine kinase inhibitor regorafenib significantly increased water diffusivity in tumorous tissue after 6 days of treatment. We also observed that regorafenib significantly reduced tumor growth compared to the control group. Using either tumor ADC changes or tumor growth to distinguish between therapy and control group resulted in diagnostic accuracy of about 78% and 83%, respectively, which we consider not sufficient for an imaging biomarker. The approach to combine both parameters using Fisher's linear discriminant analysis, substantially improved the accuracy to about 96%, thus highlighting the potential of multi-parameter MRI as an imaging biomarker for non-invasive early tumor therapy monitoring.

## Supporting Information

Table S1 **Voxelwise calculated ADC values for each animal and measurement.**
(XLSX)

Table S2 **Individual tumor ADC values for each animal and measurement.**
(XLSX)

Table S3 **Individual tumor volumes for each animal and measurement.**
(XLSX)
